# Retrospective and Prospective Analysis on “Sexting”: Indicators of Productivity, Dispersion, and Content (2009–2019)

**DOI:** 10.1007/s10508-023-02562-1

**Published:** 2023-02-27

**Authors:** Antonio Manuel Rodríguez-García, Antonio José Moreno-Guerrero, Marina García-Carmona

**Affiliations:** 1 Department of Didactics and School Organization, Faculty of Education and Sport Sciences (Melilla Campus), University of Granada, C/Santander 1, 52071 Mellila, Spain; 2 Department of Didactics and School Organization, Faculty of Education, Economics and Technology (Ceuta Campus), University of Granada, Ceuta, Spain

**Keywords:** Sexting, Sexual behavior, Adolescents, Scientometrics

## Abstract

In the last decade, research on “sexting” has undergone an exponential increase, giving rise to the publication of numerous studies clarifying its meaning, offering information of educational value, and favoring a good use of technology to prevent problems caused by this exchange of sexual information. The aim of this article was to analyze the production, performance, impact, and content of scientific articles evaluating the “sexting” thesaurus (title, abstract, and/or keywords) between 2009 and September 2019. Articles were sourced from two internationally recognized databases: Scopus and Web of Science. A scientometric study was then carried out on a sample of 641 articles that met the established inclusion criteria. The main findings indicate that “sexting” is a very recent research focus, but one in full growth phase, with scientific production related to the topic likely to double over the next few years. Although “sexting” has been researched worldwide, the scientific production of the US and American authors is the most notable. There were also some differences between the Scopus and Web of Science databases, mainly in the volume of production and the trend. However, the studies do show a common research line, “cyberbullying,” and a common target population: adolescents. Therefore, the content analysis reveals that research on “sexting” is mostly carried out with adolescents and takes into consideration other themes such as cyberbullying, dating violence, and sexuality.

## Introduction

In recent decades, development has been marked by advances in technology in the economic, social, educational, and political spheres (Espigares-Pinazo et al., 2022; Fernández-de-Álava et al., 2017). Information and Communication Technologies (ICT) open up an immense range of communication possibilities that favor and facilitate socialization processes, especially for younger users. Internet and the use of social networks (e.g., Facebook, Twitter, LinkedIn, YouTube, Instagram, Snapchat) have become a key piece in people's lives, consciously or unconsciously influencing their development (Bas Peña & Pérez de Guzmán, 2010).

As a consequence of these advances, communication forms and skills have changed and text messages, audio notes, and the exchange of audiovisuals have become central pieces in the social life of adolescents. This age generally corresponds to the age of sexual identity and exploration (Ott & Pfeiffer, 2009). During adolescence, the construction of a personal and social identity also involves the exploration of sexuality (Davis, 2013), as something that is inherently a part of everyone’s lives and marks relationships (Manning, 2013). Media use and effects on sexuality vary dramatically by a number of factors, including sexual maturity, gender, and race (Brown et al., 2009). In this process of human development, technology and the Internet, with their immediate and unlimited access to all kinds of information and with a certain sense of privacy, play a determining role (O’Sullivan, 2014). For this reason, families and teachers are increasingly concerned about the role of smartphones in the sexual lives of adolescents and young adults.

In this context, the term “sexting” arises, which is a portmanteau for “sex” and “texting.” It refers to the exchange of sexually explicit or provocative content (text messages, photographs, and videos) through a mobile phone, smartphone, Internet, or social networks (Morelli et al., 2016). One of the first published studies on “sexting” was conducted in 2009, before the current prolific use of smartphones among young people. This study indicated that 4% of 12–17 year olds with mobile phones were reported to have sent nude or semi-nude images of themselves to someone, and 15% received such images from someone they knew on their phone (Lenhart, 2009). Furthermore, a recent meta-analysis by Madigan et al. (2018) noted rates of youth-driven sexting behaviors increased over the past decade, with approximately 15 and 27% of youth (individuals 10–17 years old) sending and receiving such messages, respectively. Although sexting may occur at different ages, the study carried out by Madigan et al. revealed that the prevalence of sexting increased as youth age. An earlier study carried out by Strassberg et al. (2013) highlighted that rates from high school samples indicate even higher levels of receiving sexting content. The study by Fix et al. (2021) informs the need to integrate adolescent sexting behaviors into comprehensive sexual education curricula.

Since then, these figures have been increasing (Drouin et al., 2015; Patchin & Hinduja, 2019) and research on “sexting” has surfaced in order to clarify its meaning (Englander, 2019), to offer information of educational value (Jorgensen et al., 2019), and to favor a good use of technology to prevent problems caused by this exchange of sexual information (Madigan et al., 2018; Van Ouytsel et al., 2018).

The term shows inconsistencies in its definition depending on the research consulted, since some authors limit “sexting” to sending sexually explicit photographs or videos, while others also include sending sexually provocative text messages (Mitchell et al., 2012). A distinction is also made between “active sexting,” which refers to the sending of this material, and “passive sexting,” which has to do with the reception of sexual content (Temple & Choi, 2014). In addition, other investigations carried out by Walker et al. (2011) differentiate between consensual “sexting” (sending sexual content voluntarily) and non-consensual (when an image is used incorrectly and sent without permission), the latter being considered a form of sexual violence (Alonso & Romero, 2019). Furthermore, other research indicates that sexting may be linked to personality predictors (Alonso & Romero, 2019), to the victimization of cyberbullying and dating violence (Quesada et al., 2018), to drug use (Van Ouytsel et al., 2018), or may have consequences such as damage to reputation or even suicide (Kopecky, 2012).

For all these reasons, research into this phenomenon has increased exponentially, leading to the publication of numerous studies (Fix et al., 2021; Klettke et al., 2014; Madigan et al., 2018). Given that we are dealing with a very important subject, but at the same time so recent, this article aims to analyze the production, performance, impact, and content of scientific articles that contemplate the thesaurus “sexting” in Scopus and Web of Science in the last decade. This period coincides with the lifetime of the concept of “sexting.” Both databases were chosen because of their comprehensiveness and multidisciplinary coverage (Saif et al., 2023). The study of scientific articles as an object of analysis is very relevant as they reflect the lines, trends, and potentialities of research in universities and related institutions (Repiso et al., 2011).

## Method

This work is based on a quantitative methodology typical of scientometric studies (Aria & Cuccurullo, 2017), which makes it possible to analyze the performance and evolution of science (Todeschini & Baccini, 2016) on a given topic. Bibliometrics apply mathematical and statistical methods to scientific literature (e. g., papers, books, proceedings) and to the authors who produce it with the aim of studying and analyzing scientific activity. For this purpose, we used bibliometric laws and indicators, which measure and provide information about the results of scientific activity in its different manifestations.

According to Durieux and Gevenois (2010), there are three types of bibliometric indicators. Firstly, quantity indicators, which measure the productivity over time of a particular researcher, specific topic, geographic area, and source type (e. g., journals, book series). Secondly, quality indicators, which measure the quality (or “performance”) of a researcher's output or topic, such us journal’s impact, cites, h index, and g index. Thirdly, structural indicators, which measure connections between publications, authors, keywords, and areas of research.

Furthermore, in this research we considered the most important bibliometric laws as follows (Todeschini & Baccini, 2016):

- Law of exponential growth (Price). Price affirmed that the growth of scientific information occurs at a much higher rate than other social phenomena, although very similar to other observable facts. Price claimed that, depending on the area of knowledge, information tends to double with exponential growth every 10–15 years. He identified four phases of growth: precursors (initiation), exponential growth (boom), linear growth (maintenance), and collapse of the scientific field.
- Law of productivity of authors (Lotka). This law states that there is an unequal distribution of productivity among authors and that, regardless of the discipline, most authors publish the least number of papers, while a few authors publish most of the relevant literature on a research topic and form the most prolific group.
- Law of dispersion of scientific literature (Bradford). This law establishes that there are a number of scientific works on a given topic concentrated in a few journals that can be distributed in several concentric zones of decreasing productivity. Thus, most of the specialized literature on a topic is found in a few journals that are more prolific on a particular issue (core of scientific production), while the rest of the works would be more dispersed in different journals.

Therefore, this research had the following specific objectives: (1) to carry out a metric analysis of scientific articles indexed in Scopus and Web of Science; (2) to study diachronic, author, and geographical productivity; (3) to analyze the dispersion of scientific activity on “sexting”; and (4) to investigate the indicators of science content according to their thematic development, evolution of the scientific field, and interrelation between the main key concepts.

In order to respond to our objectives of study, we take as the only descriptor the keyword “sexting.” To obtain the units of analysis, an open search was carried out on the subject (title, abstract, and/or keywords), taking the year in which the first indexed research emerged (2009) up to September 2019 as the period of time analyzed. The selection of these two databases was made taking into account the endorsement received from the scientific community, due to the quality of their research (Garfield, 2006) that is generating a greater impact among the scientific community and evaluation agencies.

The Web of Science (Clarivate Analytics) is the collection of databases of bibliographic references and citations from periodicals that collect information from 1900 to the present. The WOS is composed of the Core Collection, which covers the Science, Social Science, and Arts and Humanities indexes, as well as the Proceedings of both Science and Social Science and Humanities along with tools for analysis and evaluation, such as the Journal Citation Report (JCR) and Essential Science Indicators. The WOS searches over 13,000 peer-reviewed journals and over 120,000 conference events in the science, social sciences, arts, and humanities. Scopus is a database of bibliographic references and citations from the Elsevier company and quality web content, with tools for monitoring, analyzing, and visualizing research. It is also the largest database of citations and abstracts of peer-reviewed literature covering about 23,000 scientific quality journals (SJR—SCImago Journal Rank), books, and conference proceedings. These two databases contain more than one million scientific references, a very representative amount for a study of this kind (Hernández-González et al., 2016; Rodríguez-García et al., 2019a, 2019b).

### Procedure

**Fig. 1:**
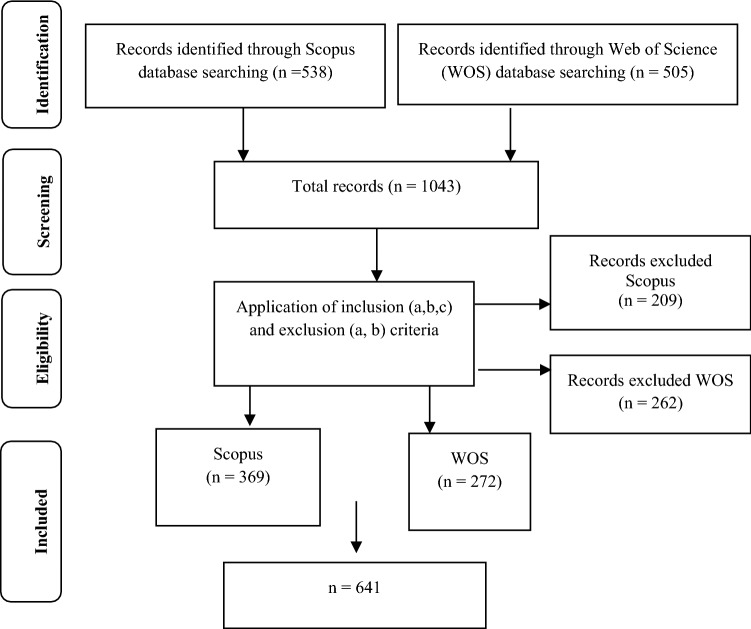
Flow diagram

The units of analysis consisted of all journal articles indexed in Scopus and in the main Web of Science collection on the topic “sexting” that have met the inclusion criteria determined (Table 1). In order to obtain Fig. 1, a search procedure was carried out according to the PRISMA protocol (Moher et al., 2009) which ended with the downloading of the data in September 2019.

**Table 1:** Inclusion and exclusion criteria

Inclusion criteria	Exclusion criteria
Journal articles	Proceedings of congresses, book chapters, or other types of publications
Indexation in the WOS indices (Journal Citation Reports): Social Science Citation Index (SSCI); Science citation index (SCI), and Arts and humanities citation index (AHCI)	Indexation in WOS indices: Emerging Sources Citation Index (ESCI); Conference Proceeding Citation Index (CPCI), and Book Citation Index (BCI)
Indexation in the SCImago journal rank (SJR) indices

### Analysis

The analysis of the data was carried out using different computer programs. In order to calculate the metric analysis, production indicators, authors' productivity, geographical, and dispersion indicators, we used SPSS v.25, Publish or Perish v.6, and Excel. In addition, we have taken the impact indicators granted by the metrics established in the two databases under study.

The content indicators were analyzed using SciMAT v.1.1.04 and VOSviewer v.1.6.13 software through the co-occurrence and co-words analysis technique (Hirsch, 2005). These indicators are based on the h index and the number of citations (Cobo et al., 2011), and it allows the development of a science map and a performance analysis to identify and represent the conceptual subdomains of the research field and their progress in the subject of study. The longitudinal co-words analysis, established with the SciMAT program (Cobo et al., 2012), was structured in four phases (Montero et al., 2018):

1. Detection of research topics: With the references used from the database Scopus (*n* = 369) and Web of Science (*n* = 272), a co-occurrence network was generated through nodes, whose base is the previously located keywords. These keywords are connected to each other when two of these words co-appear in different scientific texts, generating a normalized network of co-words through a clustering algorithm and locating the research topics, thus showing the strongly related keywords. The keywords given by the authors and those created by WoS and Scopus, according to the type of document, were the ones used. In addition, the scientific production was analyzed to check that there were no repeated documents or keywords. For doing this, all the manuscripts were visually checked using the SciMAT program, arranged in alphabetical order.
2. Representation of research theme: a strategic diagram and a thematic network (Callon et al., 1991) have been used to represent the research theme. They include two dimensions (centrality and density), where the keywords are shown in four sectors: upper right sector, where it houses the driving and fundamental themes in the research theme; upper left sector, where the connections are weak and are nodes with little relevance in the theme; lower left sector, where the themes are relevant but do not have a keen development; and lower right sector, where the nodes lack development or relevance, although those that appear in that area may be emerging themes.
3. Location of thematic areas. This was determined by the chronological evolution shown by the nodes from one period to another. The strength of the relationship was based on the number of keywords they have in common. The established periods have been 2009–2015 (P1) and 2016–2019 (P2), making this distribution in order to maintain an equitable number of words in both periods.
4. Performance analysis. Each one of the keywords, in turn, has a chain of connections that mark the trend of that node, offering data on the use that the scientific community makes of it. For that purpose, analysis protocols were established, as shown in Table 2.

**Table 2:** Protocols for the analysis

Configuration	Values
Unit of analysis	Keywords authors, keywords WoS
Data reduction	P1 (2), P2 (2)
Kind of matrix	Co-occurrence
Network reduction	P1 (2), P2 (2)
Normalization	Equivalence index
Clustering algorithm	Maximum size: 9; Minimum size: 3
Evolution map	Jaccard’s index
Overlapping map	Inclusion index

Finally, the co-occurrence analysis of keywords in the title, abstract, and keywords was carried out taking as criteria that the frequency of appearance of the concept should be x ≥ 10, and establishing a series of thematic cluster relations between different keywords.

## Results

A total of 641 articles on “sexting” were analyzed (Table 3), with 369 from Scopus and 272 from Web of Science. Scientific production begins in 2009 in the first database and in 2010 in the second. Despite being a short period of research time (10 years), the articles received a total of 10,761 citations, with an average value of more than 500 per year. The cumulative impact of scientific production (h index) calculated taking as a reference the number of years since the first publication, gives a value of 41 in Scopus and 38 in Web of Science. Giving greater weight to the literature that has received the greatest number of citations, we found the g index metric for the two databases, obtaining a value of 62 for both scientific productions.

**Table 3:** Metric analysis

Metric	Scopus	Web of science
Publication years	2009–2019	2010–2019
Citation years	10 (2009–2019)	9 (2010–2019)
Papers	369	272
Citations	5701	5024
Cites/year	570.10	558.22
Cites/paper	15.45	18.47
Cites/author	2418.03	2164.49
Papers/author	175.10	129.21
Authors/paper	2.87	2.98
h index	41	38
g index	62	62
hI, norm	25	24
hI, annual	2.50	2.67

### Production Indicators

**Fig. 2:**
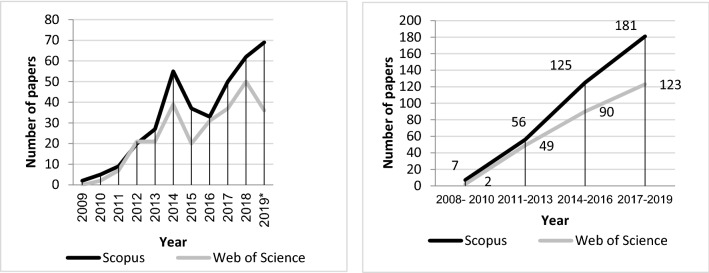
Diachronic productivity. **a** Annual productivity (2009–2019). **b** Triennial productivity (2008–2019). *Documents included up to September 2019

The analysis of diachronic productivity gives us a vision of the linear progress of the rescued scientific literature. As can be seen in Fig. 2, this is a very recent field of research, with only 10 years of scientific production and an irregular growth model (Fig. 2a). Thus, Price's law is not applicable, which establishes that after 10 years the literature will tend to double. However, grouping the publications in temporary trienniums we can observe a progressive linear growth of science in this field (Fig. 2b) with an exponential growth, being the triennium 2017–2019 the period of time that concentrates more publications. We observe, therefore, two differentiated stages: the period 2008–2010 (precursors or beginning of the research), which presents a constant linear distribution and where stability prevails, as well as little scientific production; and the period between 2011–2019, where distribution adopts a trend of increasing linear development. Therefore, according to Price, there is still a pending period in the next years for the saturation of research on this topic.

### Productivity of Authors

We find a degree of multi-authorship in both databases of *M* ≥ 2.87, so most of the articles were by three or more authors. More specifically, in Scopus there were 610 authors across the 369 papers, while in Web of Science the number of authors amounted to 603. However, according to Lotka’s law, the greatest scientific production of impact was concentrated, among five authors who were the most prolific in this respect (*x* ≥ 10log) (Table 4). We can observe that Drouin (Purdue University Fort Wayne, USA) was the most productive author in both databases. However, Temple (University Blvd Galveston, USA) was the author whose research has had the greatest impact on the scientific community, which in turn results in a higher h index in both databases.

**Table 4:** Authors with higher production and impact

Author	Institution	Country	Scopus				Web of Science			
			Doc	Quotes	Impact	*h *index	Doc	Quotes	*Impact*	*h *index
Drouin, M	Purdue University Fort Wayne	USA	13	800	61.5	15	11	329	29.9	6
Ponnet, K	Univ Ghent	Belgium	12	1180	98.3	19	10	131	13.1	6
Temple, J.R	University Blvd Galveston	USA	10	1968	196.8	24	8	288	36	5
Van Ouytsel, J	Univ Antwerp	Belgium	13	365	28.1	12	10	145	14.5	7
Walrave, M	Univ Antwerp	Belgium	13	907	69.8	16	11	163	14.8	7

### Geographical Productivity

The map of scientific research on sexting is extended to a global level (America, Asia, Europe, Africa, and Oceania). However, the core of scientific production is focused on six countries that were more prolific in this respect and, more specifically, on three specific institutions belonging to them (Table 5). Research in the USA (nS = 179 and nW = 138) obtains the highest number of citations in both databases, as well as the highest impact index in Scopus in comparison with the rest of the most productive countries. However, the analysis of the research found in WoS determined that the Canadian research (nW = 10) as a whole is the one that has generated the greatest impact on the SCI, SSCI, and AHCI indices.

**Table 5:** Most productive countries and impact of research

Country	Institution	N Scopus	Quotes	Impact	N WoS	Quotes	Impact
USA		179	3810	21.3	138	2287	16.6
	Purdue University Fort Wayne	13	367	28.2	11	329	29.9
	UT Medical Branch at Galveston	10	333	33.3	12	345	28.8
Australia		37	600	16.2	31	506	16.3
Spain		35	222	6.3	23	142	6.2
England		28	455	16.3	19	328	17.3
Belgium		21	356	17	16	258	16.1
	Universiteit Antwerpen	16	280	17.5	13	195	15
Canada		15	216	14.4	10	202	20.2

With respect to the most productive institution, the Universiteit Antwerpen (Belgium) stands out in both databases. Nevertheless, and despite having less work in this regard, the scientific community supports to a greater extent the research of the UT Medical Branch at Galveston, whose impact index is higher in Scopus (33.3), as well as the articles of the Purdue University Fort Wayne in WoS (29.9).

### Dispersion Indicators

**Fig. 3:**
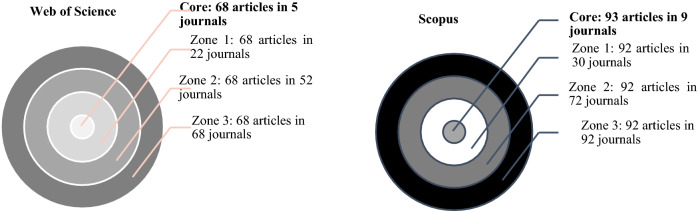
Dispersion of the scientific production WoS and Scopus

The dispersion of scientific production, which takes into account the distribution of journals based on the amount of documents published on sexting, was calculated applying Bradford's law (Fig. 3). In total, 136 journals indexed in SCI, SCCI, and AHCI have published on the subject. However, the core of the production (*x* > 5) is found in six specific journals indexed in WoS (*Computers in Human Behavior*, *Pediatrics*, *Cyberpsychology Behavior and Social Networking*, *Journal of Adolescence*, and *Journal of Adolescent Health*). At Scopus, the total number of journals is 160, with the most prolific nucleus consisting, in addition to the previous ones, of the following ones: *International Journal of Cyber Criminology*, *Sexuality and Culture,* and *Cyberpsychology* (Table 6)*.*

**Table 6:** Nucleus of scientific production

Journal	N Scopus	SJR Impact	Quotes	Impact	N WOS	JCR Impact	Quotes	Impact
Computers in Human Behavior	37	6.14	761	20.6	37	4.9	840	22.7
Pediatrics	8	5	651	81.4	9	6.4	1049	116.6
Cyberpsychology Behavior and Social Networking	9	3.12	170	18.9	9	3.9	145	16.1
Journal of Adolescence	8	2.89	201	25.1	8	2.9	175	21.9
Journal of Adolescent Health	9	4.01	460	51.1	7	5.1	434	62
International Journal of Cyber Criminology	10	2.17	42	4.2	–	–	–	–
Sexuality and Culture	8	1.26	79	9.9	–	–	–	–
Cyberpsychology	6	1.88	112	18.7	–	–	–	–

### Content Indicators

The co-words analysis carried out in the analysis units extracted from Scopus and WoS provides us with a specific view on the structural and thematic development of research and the evolution of the scientific field, as well as the main key concepts interrelated in the research.

**Fig. 4:**
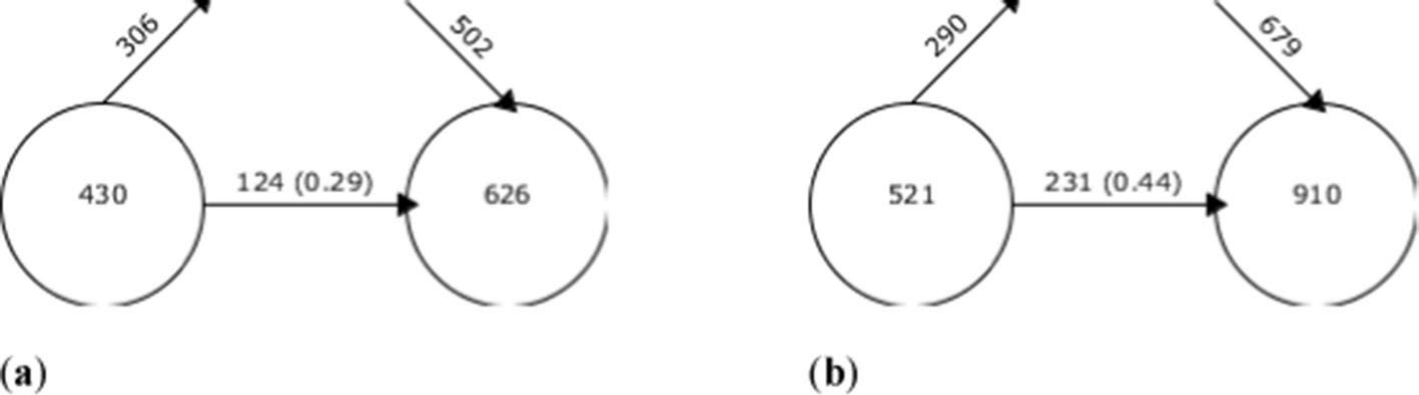
Keyword continuity between periods **a** Scopus, **b** WoS

The analysis of keyword continuity in the Scopus and WoS databases differed from each other (Fig. 4). While for the first period of analysis established (2009–2015) in Scopus, there were a total of 430 key concepts; in the second (2016–2019), this figure rises to 626. However, 306 concepts were removed from the first period with respect to the second, where 502 new words were added. Therefore, only 29% of the thesauri of the first period analyzed remain in the second period. In WoS, 521 keywords make up the first period and 910 the second, with—on this occasion—44% of the concepts of the first with respect to the second surviving.

**Fig. 5:**
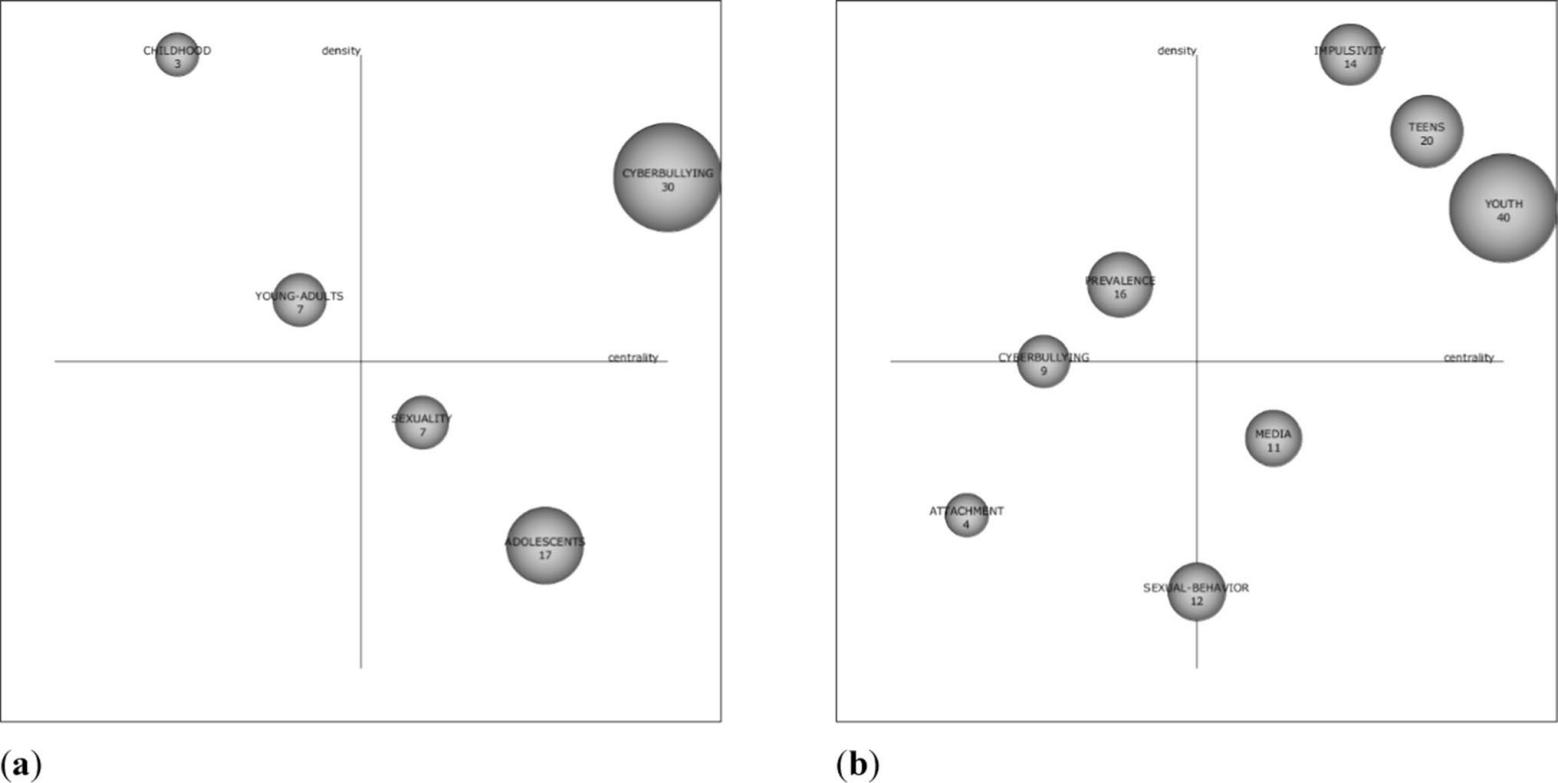
Strategic dating diagram for 2009–2015 in **a** Scopus, **b** WoS

Analyzing the strategic diagram of the first period in the Scopus and WoS databases, as shown in Fig. 5, differences are shown on the most relevant topics in both databases. In Scopus (Fig. 5a), “cyberbullying” appears as the driving theme, which is also the one that received the most citations from the scientific community, whose research focuses on adolescents, internet safety, social networks, cyberwords, “sexting,” bullying, internet, and harassment. In this period, the thesaurus “adolescents,” although it appears as a basic and transversal theme, continues to be of relevance to the scientific community, given its high level of citation and its focus on studies on sexual risk, explicit images on mobile phones, communication via mobile phones, sexual behavior, risk behavior, mobile phones, feelings of disgust, and “sexting” in adolescents. In addition, it is relevant that no topic appears in the emerging zone.

In WoS, the perspective changes completely (Fig. 5b). The driving themes were “impulsivity,” which refers to feelings of disgust, adjustment rates, “sexting” in adolescents, performances, adolescents, personality, school students, and alcohol use. In addition, we find the term “teens,” which focuses on young people's actions, explicit images of mobile phones, adolescents, risky behavior, mobile phones, opportunities, and the internet; and “youth,” which relates to popularity, performance evaluation, sexuality, sexting, internet, testing, teen exposure, and explicit internet material. It is also observed that “prevalence,” although a very developed and isolated topic, remains relevant to the scientific community, which focuses its research on adolescent girls, substance use, telephones, petitions, risk, mental health, predictors, and sexual risk. In this period, “attachment” appears as an unknown topic, dealing with research on university students, consent, and women.

**Fig. 6:**
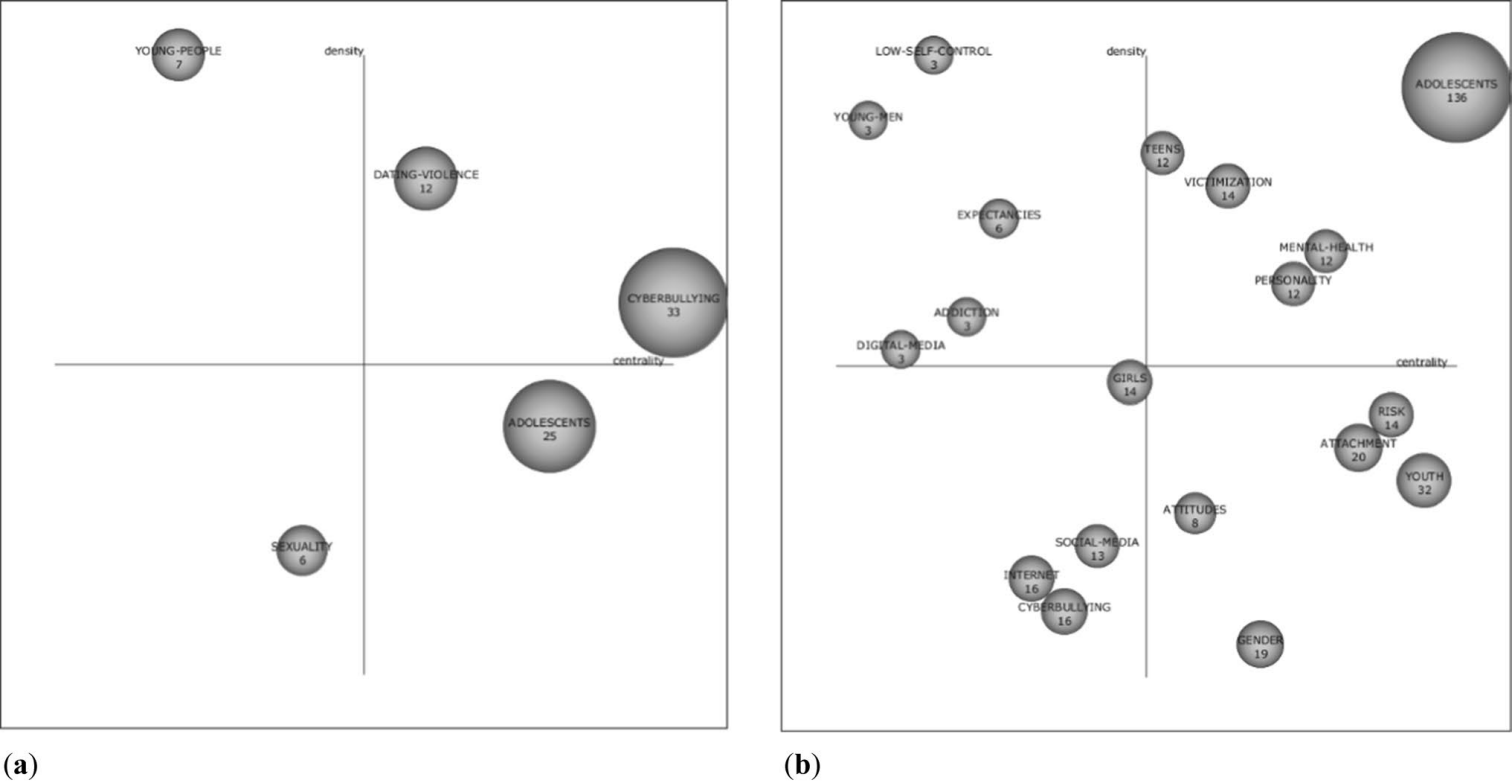
**a** Strategic appointment diagram for 2016–2019 in Scopus. **b** Strategic appointment diagram for 2016–2019 in WoS

In the second period established (Fig. 6), significant differences were again observed in both databases. In Scopus (Fig. 6a), the driving themes were “dating violence,” which relates to digital contact abuse, domestic violence, victims, sexual extortion, internet and abuse, sexual assault, young adults, and sexual harassment; and “cyberbullying,” which focuses its research on friendship, interventions, obscenity, minors, schools, “sexting,” harassment, and sexual harassment. Although situated as a basic and cross-cutting theme, “adolescents” remains relevant to the scientific community, given its high level of citation. In this case, the research relates to validation, problematic uses of the internet, explicit sexual material, gender, self-representation, social networks, qualitative research, and sexual development. In this period, “sexuality” appears as an unknown topic, which should be taken into account, given that in the coming years, in the Scopus database, it may be a driving theme. Its studies were associated with the internet, young people, and photographs of the male genitals.

In WoS (Fig. 6b), the perspective changes considerably. There were five driving themes. Firstly, “adolescents,” which was the most cited topic and was related to prevalence, sexting, predictors, sexual behavior, association, behavior, risk behavior, and adolescents. Secondly, “teens,” which was associated with rape, childhood, effects, images, risky behavior, aggression, consent, and sexual abuse of adults. We found, in turn, “victimization,” which focused on interpersonal relationships, college students, violent youth abuse, violent relationships, internet and abuse, sexual assault, perpetration, and adolescent victims. “Mental health,” whose research focused on self-objective, sociocultural attitudes, public health, experiences, women, psychological distress, depression, and homosexuality. Finally, “personality,” oriented to self-esteem, individual differences, adults, models, online, and impulsivity.

On the other hand, it is necessary to emphasize the theme “youth” that, although it appears as a basic and transversal theme, presents a high number of citations, being related to disclosure, abuse, youth, technology, violence, victims on the net, pornography, and sexual exploration of young people. In this period, four themes appear in the lower left quadrant, which should be kept in mind for the coming years, as they may be driving themes. Thus, we find the term “girls,” which relates to boys, the media, friendships, masculinity, conversation, femininity, and sexuality; “social media,” which is associated with perceptions, qualitative research, opinions, self-representation, cyber, education, and Snapchat; “Internet,” which focuses on offline, video games, disorder, sexual activity, program, security, HIV risk, and students; and “cyberbullying,” which focuses on sexual harassment, questionnaires, Facebook, schools, minors, social networks, interventions, and harassment.

The information in Figs. 5 and 6 is reflected in Table 7, which shows the position of the different themes, as well as the density and centrality values. In this case, we find that the themes teens, youth, cyberbullying, and attachment are repeated in WoS for the two periods. The same thing occurs with the themes cyberbullying, sexuality, and adolescents in Scopus.

**Table 7:** Principal research themes related to sexting from 2009 to 2019

Theme	P1 (2009–2015)	P2 (2016–2019)
*Web of science*		
Impulsivity	Q1 (48.14/56.88)	
Teens	Q1 (49.81/33.78)	Q1 (32.79/27.38)
Youth	Q1 (73.67/22.43)	Q4 (83.67/8.39)
Prevalence	Q2 (21.54/15.45)	
Cyberbullying	Q2-Q3 (19.21/14.66)	Q3 (22/5.29)
Attachment	Q3 (12.61/13.31)	Q4 (51.17/11.47)
Sexual behavior	Q3-Q4 (22.93/8.92)	
Media	Q4 (41.07/12.42)	
Adolescents		Q1 (192.78/46.68)
Mental health		Q1 (39.13/19.42)
Personality		Q1 (37.73/18.71)
Victimization		Q1 (33.73/24.93)
Low self-control		Q2 (4.26/70.83)
Young men		Q2 (1.12/33.33)
Expectancies		Q2 (19.6/20.18)
Addiction		Q2 (15.59/18.52)
Digital media		Q2 (2.24/18.52)
Girls		Q3 (27.49/15.18)
Social media		Q3 (22.07/6.35)
Internet		Q3 (19.8/5.33)
Attitudes		Q4 (32.86/6.69)
Risk		Q4 (59.97/14.85)
Gender		Q4 (35.78/4.58)
*Scopus*		
Cyberbullying	Q1 (25.6/17.26)	Q1 (22.4/7.12)
Childhood	Q2 (1.77/33.33)	
Young adults	Q2 (4.69/11.67)	
Sexuality	Q4 (9.04/7.84)	Q3 (7.14/4.54)
Adolescents	Q4 (18.74/6.77)	Q4 (18.14/5.07)
Dating violence		Q1 (10.19/17.85)
Young people		Q2 (5.05/35.68)

As stated, the differences between the databases stem from the number of documents on the themes and the variety of terminologies used. However, as can be seen, cyberbullying themes are repeated in both databases. These differences indicate the research trends and type of data collection in each of the databases.

**Fig. 7:**
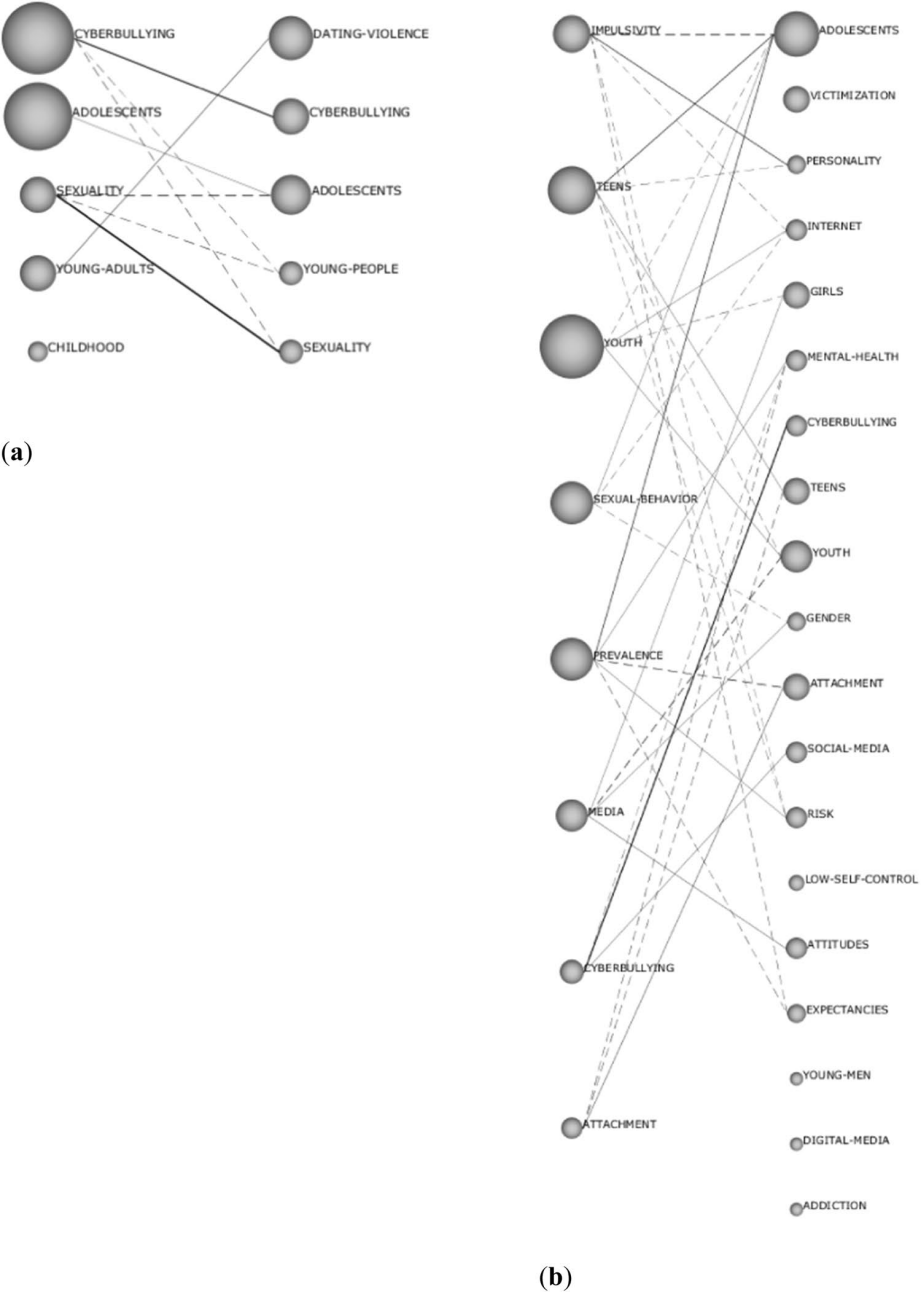
**a** Thematic evolution by index h in Scopus, **b** thematic evolution by index h in WoS

In order to understand the thematic evolution in the field of knowledge, it must be kept in mind that the connections established through solid lines determine a conceptual and thematic relation in the analyzed research, whereas the dashed lines show non-conceptual relations between the topics, sharing only keywords among them. The conceptual relationship is established when both subjects share a common theme. Non-conceptual relationships are when they only share keywords. The difference between theme and keyword lies in the appearance or not of the different established diagrams. If it appears in the diagrams, they are thematic; otherwise, they are keywords. Furthermore, the thicker the line, the stronger the existing relationship (Moreno, 2019; Rodríguez-García et al., 2019a, 2019b). Thus, if the thematic evolution of the Scopus and WoS databases is compared, as shown in Fig. 7, different trends and relationships can be observed. In Scopus, there is a thematic evolution, centered on “cyberbullying,” “adolescents,” and “sexuality,” which shows that this database maintains the three terms as the central axes of its research. The connections between them are conceptual, with greater strength in the relationship established by “sexuality” (Fig. 7a). It is also observed that, in the first period, the themes with the highest h index are “cyberbullying” and “adolescents,” while in the second period they are “dating violence” and “adolescents.”

In WoS (Fig. 7b), the perspective changes, given that in the same search conditions, more themes arise and the connections are more varied. Thematic continuity is shown between periods, centered on the themes “attachment,” “cyberbullying,” this being the strongest connection established between them, and “teens.” In addition, it is observed how the theme evolves from one period to another, emerging new themes and connections that give a glimpse of future trends in the field. Moreover, the links generated at a conceptual level between “cyberbullying,” from the first period with “social media”; between “media,” from the first period with “attitudes” and “girls”; and between “prevalence,” from the first period with “risk,” “mental health,” and “adolescents.” In the first period, the theme with the highest index h is “youth” and in the second period “adolescents.”

**Fig. 8:**
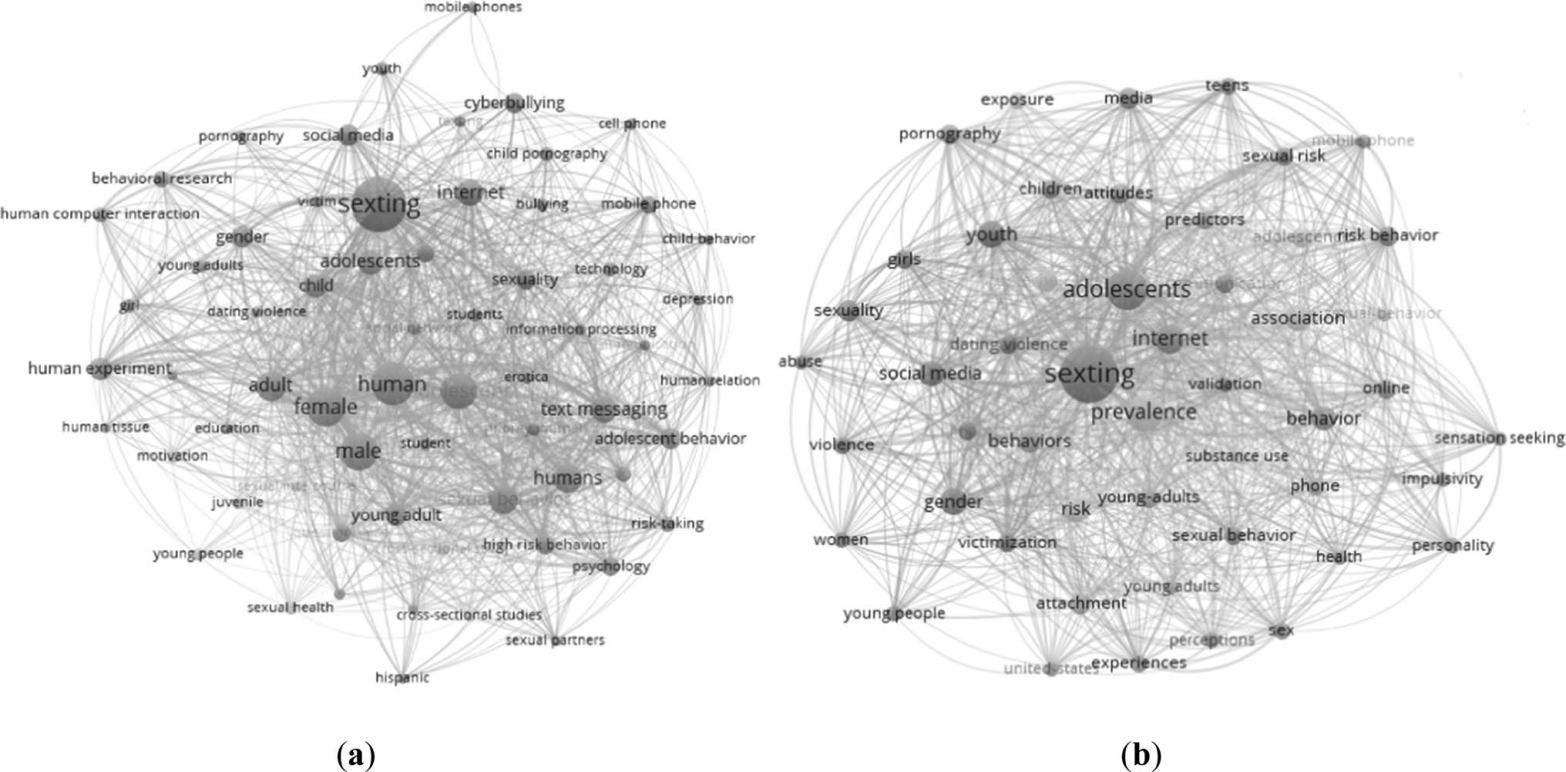
Analysis of co-occurrence of keywords. **a** Analysis of co-occurrences in Scopus. **b** Analysis of co-occurrences in WoS

Finally, the analysis of the content of the titles, abstract, and keywords granted by both the database and the authors applying a co-occurrence technique of words (*x* ≥ 10), places the thesaurus “sexting” as the central axis of the research, with a frequency of *x* = 275 in Scopus and a total of 1911 links to other concepts and *x* = 164 in WoS and with 695 links (Fig. 8).

## Discussion

After analyzing the production, performance, impact, and content of scientific articles on sexting in the Scopus and Web of Science databases, we can underline that we are facing a very recent research focus, but in full growth phase. The analyses carried out determine that scientific production on “sexting” will tend to double over the next few years in line with Price’s law. In fact, most of the current research was published during the last three years analyzed (2017–2019). The rise of smartphones and the possibility of being permanently connected to networks make it necessary to hold a debate to make a critical and ethical use of them, since we are facing a problem that is increasing (Drouin et al., 2015; Patchin & Hinduja, 2019) and that has dire consequences for subjects who suffer this type of digital violence (Alonso & Romero, 2019; Kopecky, 2012). At the same time, it should be noted that “sexting” does have a range of prosocial benefits. Holmes et al. (2021) highlight a number of these benefits, for instance increased self-esteem, sexual gratification, and increased intimacy and trust with a partner. Nevertheless, as observed in the thematic analysis, themes interrelated with “sexting” tend to be more connected to the negative aspects of the phenomenon, such as cyberbullying or violence.

There is no doubt, therefore, that research will tend to grow due to the characteristics of the society in which we find ourselves. Few authors are currently the most prolific in this regard, with only five researchers who have contributed with more than 10 papers. In addition, the main focus of scientific production is in North America (USA and Canada), followed by a notable presence of Australia and European countries (Belgium, England, and Spain). These data situate the research of sexting as a pioneering topic, of growing interest and for which more research is needed, since the analyses carried out concentrate the data on a reduced number of time, countries, authors, institutions, journals, and specific keywords.

This study also shows how the Scopus and Web of Science databases, although they collect the most relevant studies with the greatest impact on the subject under study, present differences between the two, given that they do not collect the same amount of documents or the same productions, something that must be borne in mind for future studies on this subject. Furthermore, the analysis of the continuity of the keywords between the two periods (2009–2015 and 2016–2019) shows change and reorientation in the research carried out on “sexting” from its beginnings to the present. As a consequence, the field keeps expanding, giving rise to new variables and concepts that affect this practice and which must be taken into consideration.

The same applies to the thematic analysis carried out in the two databases, given that cyberbullying is positioned as the driving theme in Scopus (2009–2015), while in WoS the central themes are impulsivity, teens, and youth. In turn, in the second period established, Scopus establishes “dating,” “violence,” and “cyberbullying” as driving themes, while WoS establishes “adolescents,” “teens,” “victimization,” “mental health,” and “personality” as driving themes.

At the same time, the thematic evolution and, therefore, the line of research recorded in both databases vary, although there is a common line of research: “cyberbullying,” which prevails for its durability and relationship with the topic analyzed (Quesada et al., 2018). In Scopus, the thematic evolution also focuses on “adolescents” and “sexuality,” while in WoS it focuses on “attachment” and “teens.” It is important to note that searches carried out in both databases with the descriptor "sexting" have resulted in the majority of research focusing on adolescents. In this way, most of the research places children and adolescents at risk when carrying out this practice, whether consented to or manifested as a form of digital violence.

Therefore, it can be concluded that, in both databases, cyberbullying is present in all the established time diagrams, which shows its relevance and interest to the field of sexting. Although this is the backbone of both databases, the trends vary from one to another. In WoS, the focus is on impulsiveness, young people, mental health, personality, and victimization. In Scopus, the focus is on the violence that can be generated in the couple. As can be seen, the trends and lines of research are different. This is relevant given that it allows researchers to know which database to select according to their analysis needs. The emerging themes and concepts of this study highlight the importance of educational intervention in relation to sexting. For this, it is necessary to train teachers and families on the critical use of ICTs and the main problems arising from their inappropriate use (García-Carmona, 2015), as well as to promote a good development of digital citizen competence, which is essential to develop effectively in today's society (Fernández-de-Álava et al., 2017; Rodríguez-García et al., 2019a, 2019b).

Another aspect to highlight is the sexual education that adolescents receive throughout their lives, both in their school and in their family context. It is important to analyze the type of relationships that are established with their peers from infancy to adolescence in order to know whether they are based on values of equality, tolerance or whether, on the contrary, they focus on sexist and/or aggressive behavior. These facts will be decisive when dealing with their social relationships through the use of smartphones and when facing a situation of “sexting.”

In this scenario, family involvement is the keystone for the management of possible conflicts produced by the misuse of technology when “sexting” occurs. Recommendations for families include teaching adolescents about the possibility of exposure to sexual images, and what action to take in such situations, as well as media literacy (Ghorashi, 2019). It is also possible to add indications related to an adequate sexual education and the promotion of values of equality within the family environment.

### Limitations of the Study

The limitations of research can focus on the few scientific texts, compared to other subjects, in all their scientific production, in addition to organizing the co-occurrence network, given the spelling mistakes found in many of the keywords of the Scopus and WoS databases. Similarly, as in studies of this nature, data may have been lost from articles that did not contemplate the keyword that guided this work.

As a future line of research, the same research can be carried out compiling the data from Google Scholar or Scielo, given that the databases of Scopus and Wos are more oriented to the Anglo-Saxon world, while the other also covers the Ibero-American area. In addition, other types of digital violence, such as grooming (Wolf & Pruitt, 2019), sextortion (Wolak et al., 2018), happy slapping (Best, 2019), etc., which are increasingly present in our society and have harmful consequences for the psychosocial development of children, adolescents, and adults, need to be taken into account.
